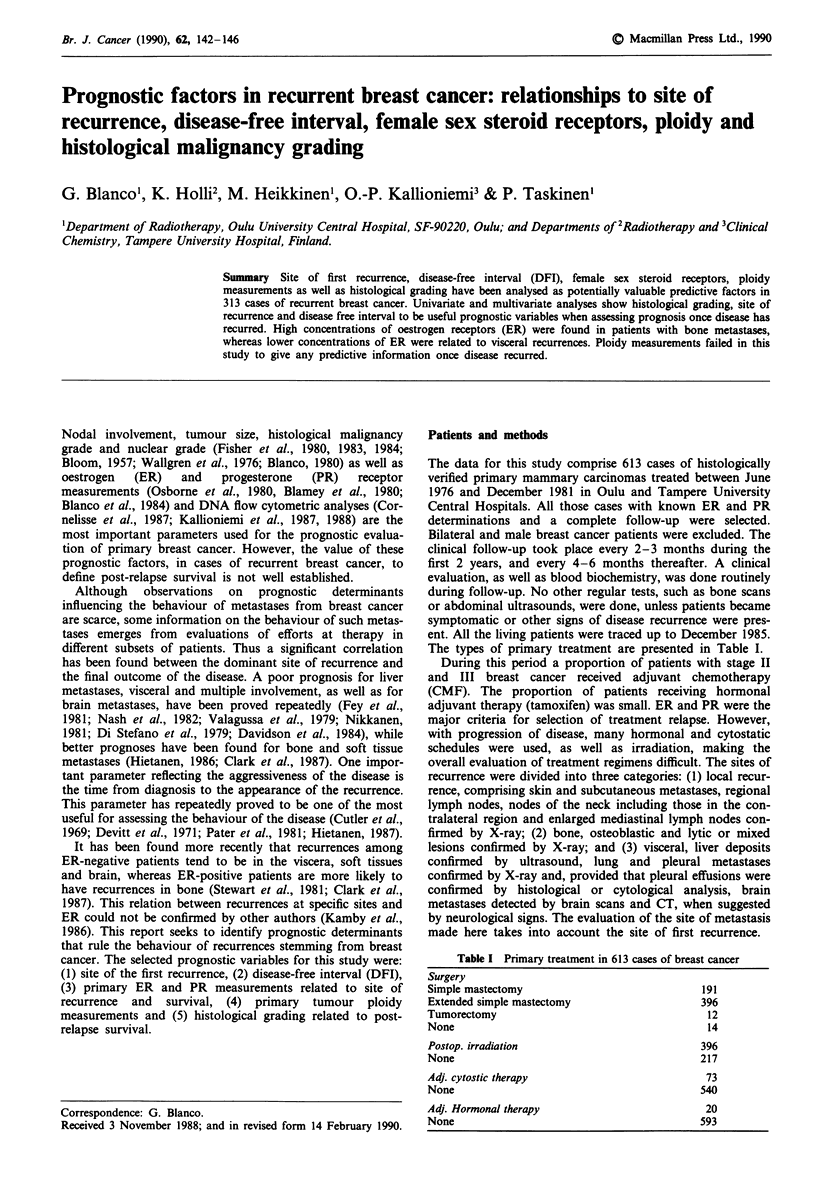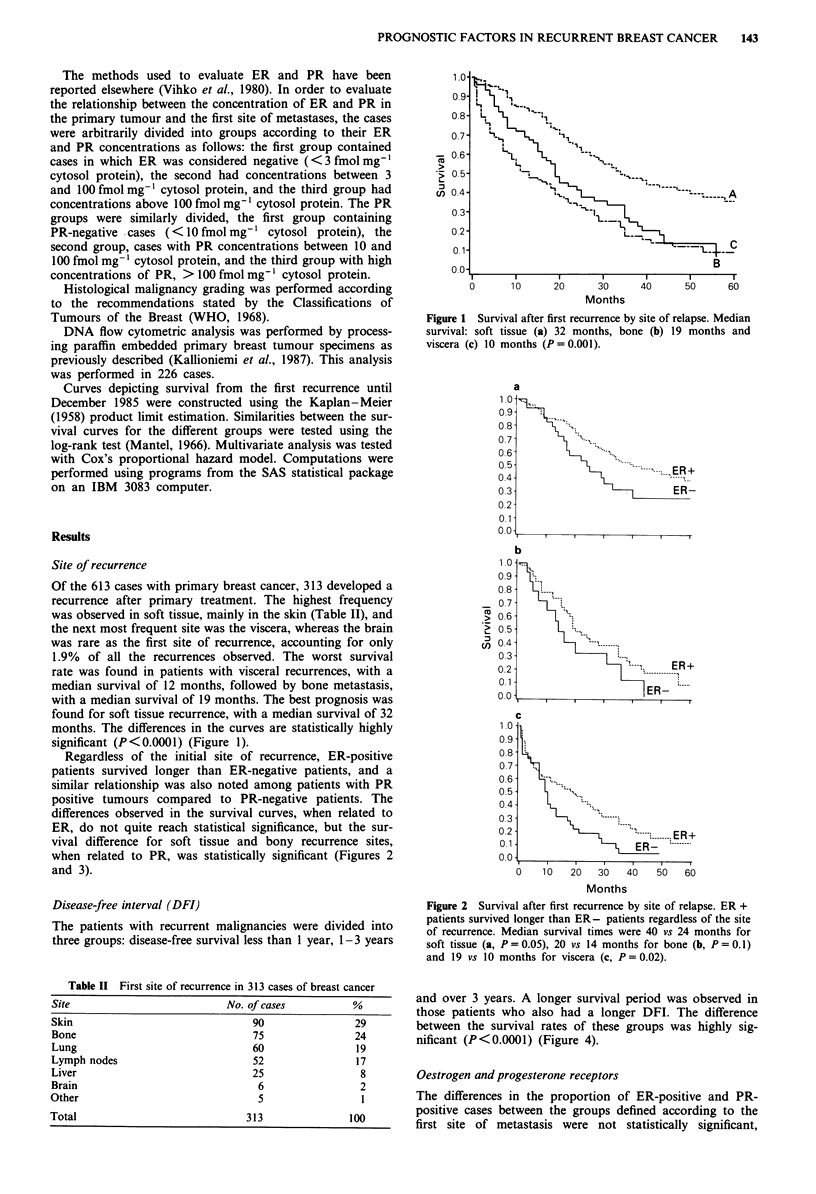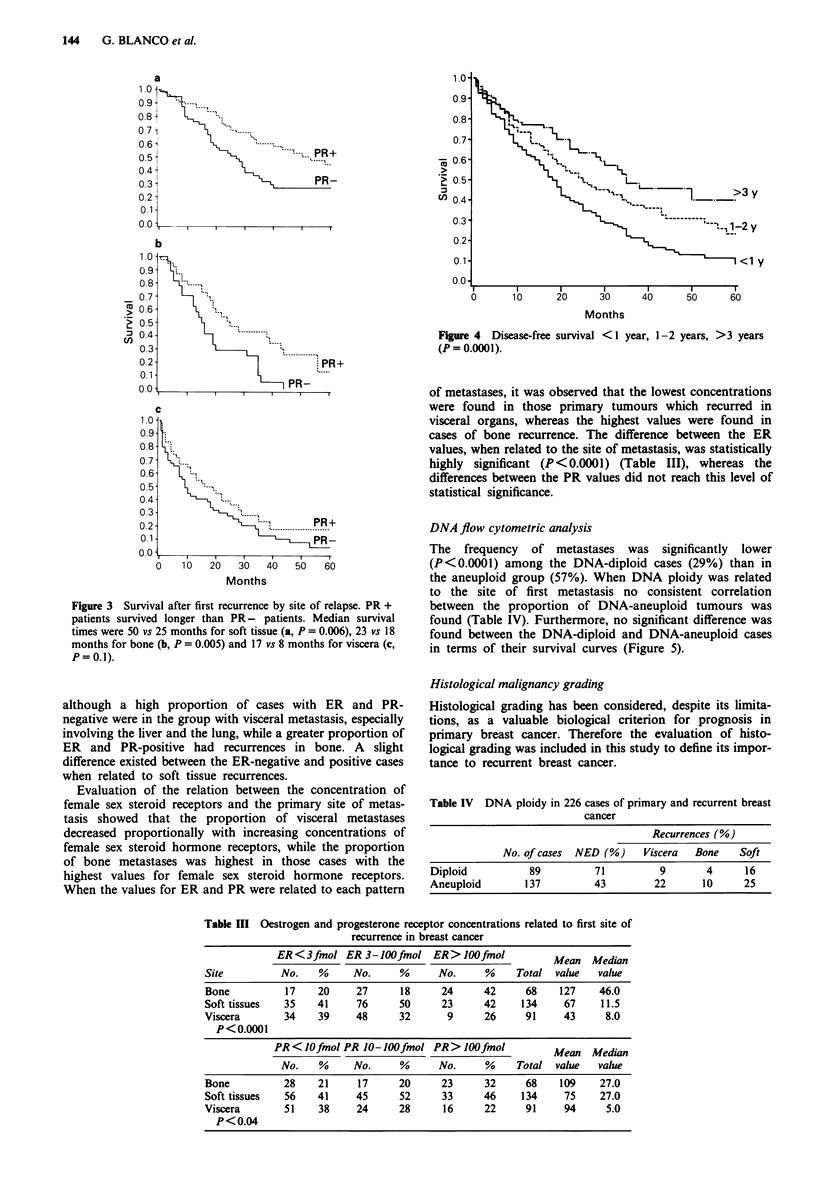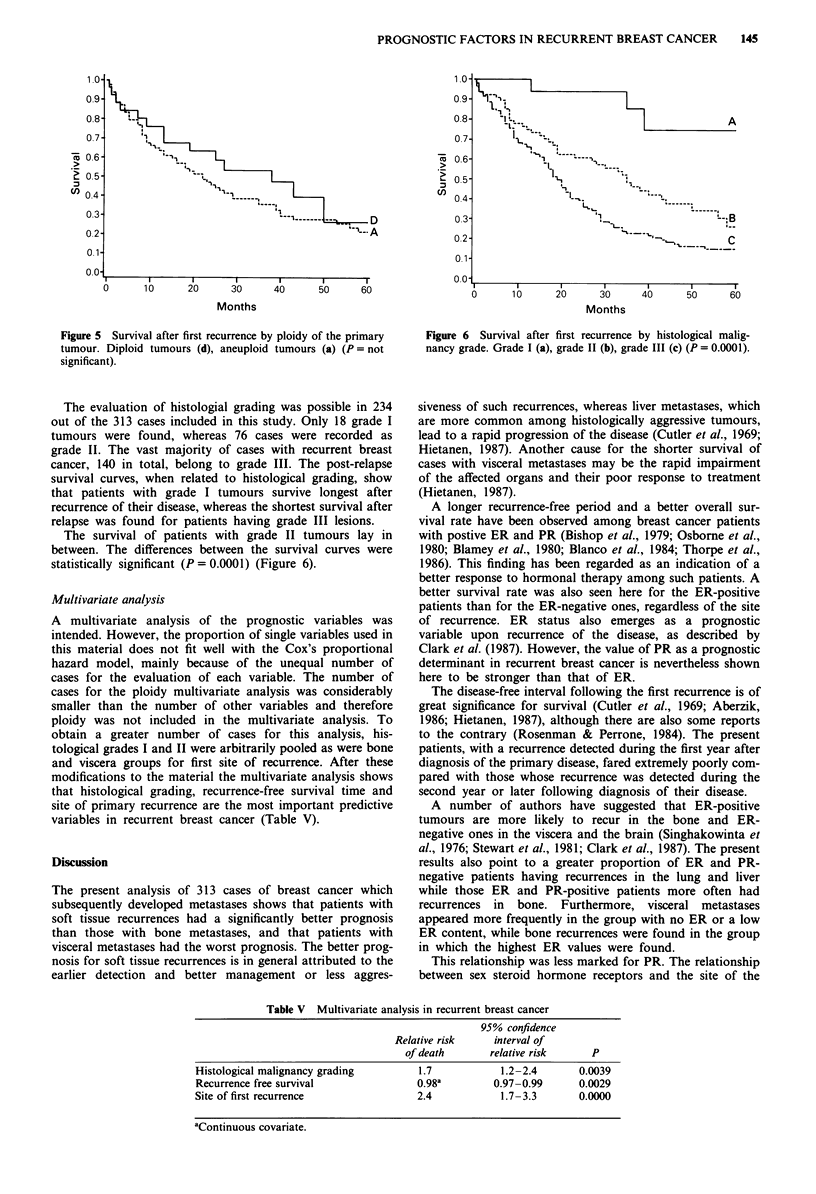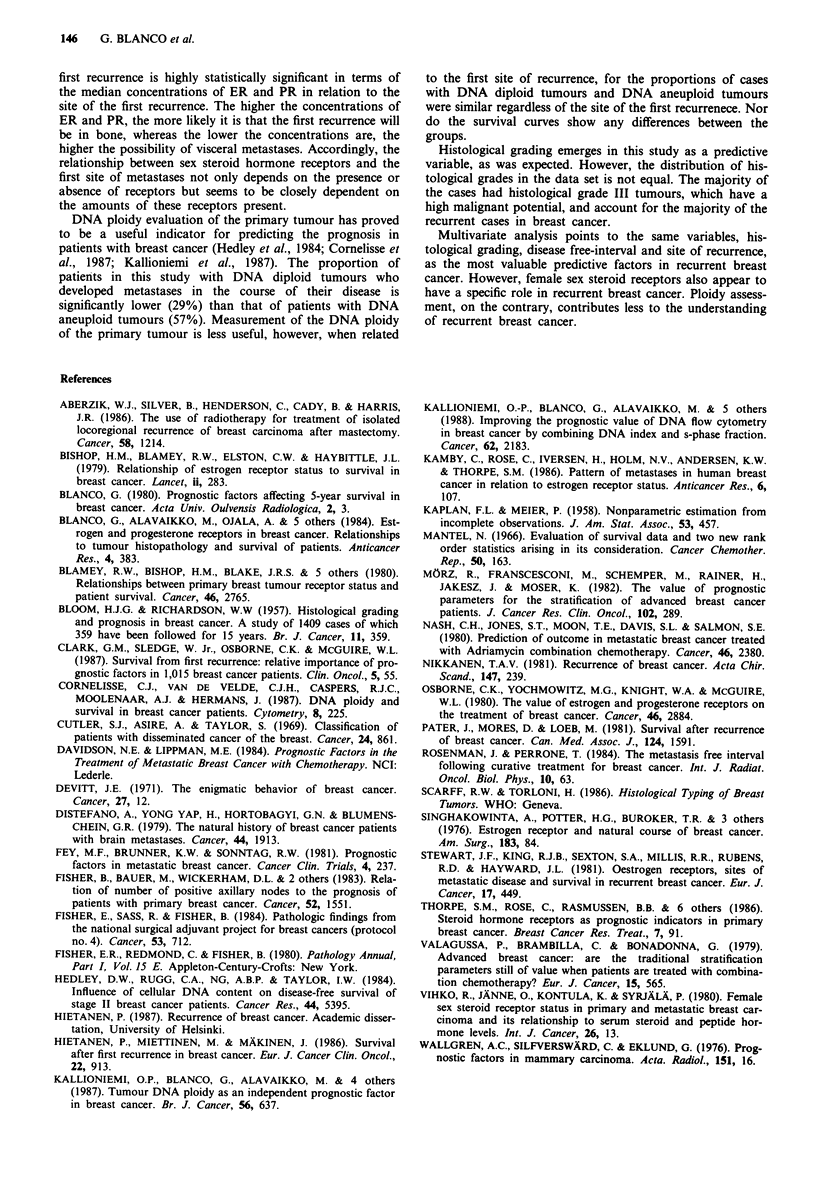# Prognostic factors in recurrent breast cancer: relationships to site of recurrence, disease-free interval, female sex steroid receptors, ploidy and histological malignancy grading.

**DOI:** 10.1038/bjc.1990.247

**Published:** 1990-07

**Authors:** G. Blanco, K. Holli, M. Heikkinen, O. P. Kallioniemi, P. Taskinen

**Affiliations:** Department of Radiotherapy, Oulu University Central Hospital, Finland.

## Abstract

Site of first recurrence, disease-free interval (DFI), female sex steroid receptors, ploidy measurements as well as histological grading have been analysed as potentially valuable predictive factors in 313 cases of recurrent breast cancer. Univariate and multivariate analyses show histological grading, site of recurrence and disease free interval to be useful prognostic variables when assessing prognosis once disease has recurred. High concentrations of oestrogen receptors (ER) were found in patients with bone metastases, whereas lower concentrations of ER were related to visceral recurrences. Ploidy measurements failed in this study to give any predictive information once disease recurred.


Br. J. Cancer (1990), 62, 142-146 © Macmillan Press Ltd., 1990

### Prognostic factors in recurrent breast cancer: relationships to site of recurrence, disease-free interval, female sex steroid receptors, ploidy and histological malignancy grading

G. Blanco[1], K. Holli[2], M. Heikkinen[1], O.-P. Kallioniemi[3] & P. Taskinen[1]

[1]Department of Radiotherapy, Oulu University Central Hospital, SF-90220, Oulu; and Departments of [2]Radiotherapy and [3]Clinical Chemistry, Tampere University Hospital, Finland.

**Summary** Site of first recurrence, disease-free interval (DFI), female sex steroid receptors, ploidy measurements as well as histological grading have been analysed as potentially valuable predictive factors in 313 cases of recurrent breast cancer. Univariate and multivariate analyses show histological grading, site of recurrence and disease free interval to be useful prognostic variables when assessing prognosis once disease has recurred. High concentrations of oestrogen receptors (ER) were found in patients with bone metastases, whereas lower concentrations of ER were related to visceral recurrences. Ploidy measurements failed in this study to give any predictive information once disease recurred.

Nodal involvement, tumour size, histological malignancy grade and nuclear grade (Fisher *et al.*, 1980, 1983, 1984; Bloom, 1957; Wallgren *et al.*, 1976; Blanco, 1980) as well as oestrogen (ER) and progesterone (PR) receptor measurements (Osborne *et al.*, 1980, Blamey *et al.*, 1980; Blanco *et al.*, 1984) and DNA flow cytometric analyses (Cornelisse *et al.*, 1987; Kallioniemi *et al.*, 1987, 1988) are the most important parameters used for the prognostic evaluation of primary breast cancer. However, the value of these prognostic factors, in cases of recurrent breast cancer, to define post-relapse survival is not well established.

Although observations on prognostic determinants influencing the behaviour of metastases from breast cancer are scarce, some information on the behaviour of such metastases emerges from evaluations of efforts at therapy in different subsets of patients. Thus a significant correlation has been found between the dominant site of recurrence and the final outcome of the disease. A poor prognosis for liver metastases, visceral and multiple involvement, as well as for brain metastases, have been proved repeatedly (Fey *et al.*, 1981; Nash *et al.*, 1982; Valagussa *et al.*, 1979; Nikkanen, 1981; Di Stefano *et al.*, 1979; Davidson *et al.*, 1984), while better prognoses have been found for bone and soft tissue metastases (Hietanen, 1986; Clark *et al.*, 1987). One important parameter reflecting the aggressiveness of the disease is the time from diagnosis to the appearance of the recurrence. This parameter has repeatedly proved to be one of the most useful for assessing the behaviour of the disease (Cutler *et al.*, 1969; Devitt *et al.*, 1971; Pater *et al.*, 1981; Hietanen, 1987).

It has been found more recently that recurrences among ER-negative patients tend to be in the viscera, soft tissues and brain, whereas ER-positive patients are more likely to have recurrences in bone (Stewart *et al.*, 1981; Clark *et al.*, 1987). This relation between recurrences at specific sites and ER could not be confirmed by other authors (Kamby *et al.*, 1986). This report seeks to identify prognostic determinants that rule the behaviour of recurrences stemming from breast cancer. The selected prognostic variables for this study were: (1) site of the first recurrence, (2) disease-free interval (DFI), (3) primary ER and PR measurements related to site of recurrence and survival, (4) primary tumour ploidy measurements and (5) histological grading related to post-relapse survival.

Correspondence: G. Blanco.
Received 3 November 1988; and in revised form 14 February 1990.

### Patients and methods

The data for this study comprise 613 cases of histologically verified primary mammary carcinomas treated between June 1976 and December 1981 in Oulu and Tampere University Central Hospitals. All those cases with known ER and PR determinations and a complete follow-up were selected. Bilateral and male breast cancer patients were excluded. The clinical follow-up took place every 2–3 months during the first 2 years, and every 4–6 months thereafter. A clinical evaluation, as well as blood biochemistry, was done routinely during follow-up. No other regular tests, such as bone scans or abdominal ultrasounds, were done, unless patients became symptomatic or other signs of disease recurrence were present. All the living patients were traced up to December 1985. The types of primary treatment are presented in Table I.

During this period a proportion of patients with stage II and III breast cancer received adjuvant chemotherapy (CMF). The proportion of patients receiving hormonal adjuvant therapy (tamoxifen) was small. ER and PR were the major criteria for selection of treatment relapse. However, with progression of disease, many hormonal and cytostatic schedules were used, as well as irradiation, making the overall evaluation of treatment regimens difficult. The sites of recurrence were divided into three categories: (1) local recurrence, comprising skin and subcutaneous metastases, regional lymph nodes, nodes of the neck including those in the contralateral region and enlarged mediastinal lymph nodes confirmed by X-ray; (2) bone, osteoblastic and lytic or mixed lesions confirmed by X-ray; and (3) visceral, liver deposits confirmed by ultrasound, lung and pleural metastases confirmed by X-ray and, provided that pleural effusions were confirmed by histological or cytological analysis, brain metastases detected by brain scans and CT, when suggested by neurological signs. The evaluation of the site of metastasis made here takes into account the site of first recurrence.

**Table I** Primary treatment in 613 cases of breast cancer

| | |
|---|---|
| *Surgery* | |
| Simple mastectomy | 191 |
| Extended simple mastectomy | 396 |
| Tumorectomy | 12 |
| None | 14 |
| *Postop. irradiation* | 396 |
| None | 217 |
| *Adj. cytostic therapy* | 73 |
| None | 540 |
| *Adj. Hormonal therapy* | 20 |
| None | 593 |

The methods used to evaluate ER and PR have been reported elsewhere (Vihko *et al.*, 1980). In order to evaluate the relationship between the concentration of ER and PR in the primary tumour and the first site of metastases, the cases were arbitrarily divided into groups according to their ER and PR concentrations as follows: the first group contained cases in which ER was considered negative ($<3$ fmol mg$^{-1}$ cytosol protein), the second had concentrations between 3 and 100 fmol mg$^{-1}$ cytosol protein, and the third group had concentrations above 100 fmol mg$^{-1}$ cytosol protein. The PR groups were similarly divided, the first group containing PR-negative cases ($<10$ fmol mg$^{-1}$ cytosol protein), the second group, cases with PR concentrations between 10 and 100 fmol mg$^{-1}$ cytosol protein, and the third group with high concentrations of PR, $>100$ fmol mg$^{-1}$ cytosol protein.

Histological malignancy grading was performed according to the recommendations stated by the Classifications of Tumours of the Breast (WHO, 1968).

DNA flow cytometric analysis was performed by processing paraffin embedded primary breast tumour specimens as previously described (Kallioniemi *et al.*, 1987). This analysis was performed in 226 cases.

Curves depicting survival from the first recurrence until December 1985 were constructed using the Kaplan–Meier (1958) product limit estimation. Similarities between the survival curves for the different groups were tested using the log-rank test (Mantel, 1966). Multivariate analysis was tested with Cox's proportional hazard model. Computations were performed using programs from the SAS statistical package on an IBM 3083 computer.

### Results

### *Site of recurrence*

Of the 613 cases with primary breast cancer, 313 developed a recurrence after primary treatment. The highest frequency was observed in soft tissue, mainly in the skin (Table II), and the next most frequent site was the viscera, whereas the brain was rare as the first site of recurrence, accounting for only 1.9% of all the recurrences observed. The worst survival rate was found in patients with visceral recurrences, with a median survival of 12 months, followed by bone metastasis, with a median survival of 19 months. The best prognosis was found for soft tissue recurrence, with a median survival of 32 months. The differences in the curves are statistically highly significant ($P<0.0001$) (Figure 1).

Regardless of the initial site of recurrence, ER-positive patients survived longer than ER-negative patients, and a similar relationship was also noted among patients with PR positive tumours compared to PR-negative patients. The differences observed in the survival curves, when related to ER, do not quite reach statistical significance, but the survival difference for soft tissue and bony recurrence sites, when related to PR, was statistically significant (Figures 2 and 3).

### *Disease-free interval (DFI)*

The patients with recurrent malignancies were divided into three groups: disease-free survival less than 1 year, 1–3 years

**Figure 1** Survival after first recurrence by site of relapse. Median survival: soft tissue (**a**) 32 months, bone (**b**) 19 months and viscera (c) 10 months ($P=0.001$).

**Figure 2** Survival after first recurrence by site of relapse. ER + patients survived longer than ER – patients regardless of the site of recurrence. Median survival times were 40 *vs* 24 months for soft tissue (**a**, $P=0.05$), 20 *vs* 14 months for bone (**b**, $P=0.1$) and 19 *vs* 10 months for viscera (c, $P=0.02$).

**Table II** First site of recurrence in 313 cases of breast cancer

| *Site* | *No. of cases* | *%* |
|---|---|---|
| Skin | 90 | 29 |
| Bone | 75 | 24 |
| Lung | 60 | 19 |
| Lymph nodes | 52 | 17 |
| Liver | 25 | 8 |
| Brain | 6 | 2 |
| Other | 5 | 1 |
| Total | 313 | 100 |

and over 3 years. A longer survival period was observed in those patients who also had a longer DFI. The difference between the survival rates of these groups was highly significant ($P<0.0001$) (Figure 4).

### *Oestrogen and progesterone receptors*

The differences in the proportion of ER-positive and PR-positive cases between the groups defined according to the first site of metastasis were not statistically significant,

**Figure 3** Survival after first recurrence by site of relapse. PR + patients survived longer than PR− patients. Median survival times were 50 vs 25 months for soft tissue (a, $P = 0.006$), 23 vs 18 months for bone (b, $P = 0.005$) and 17 vs 8 months for viscera (c, $P = 0.1$).

**Figure 4** Disease-free survival <1 year, 1–2 years, >3 years ($P = 0.0001$).

although a high proportion of cases with ER and PR-negative were in the group with visceral metastasis, especially involving the liver and the lung, while a greater proportion of ER and PR-positive had recurrences in bone. A slight difference existed between the ER-negative and positive cases when related to soft tissue recurrences.

Evaluation of the relation between the concentration of female sex steroid receptors and the primary site of metastasis showed that the proportion of visceral metastases decreased proportionally with increasing concentrations of female sex steroid hormone receptors, while the proportion of bone metastases was highest in those cases with the highest values for female sex steroid hormone receptors. When the values for ER and PR were related to each pattern of metastases, it was observed that the lowest concentrations were found in those primary tumours which recurred in visceral organs, whereas the highest values were found in cases of bone recurrence. The difference between the ER values, when related to the site of metastasis, was statistically highly significant ($P < 0.0001$) (Table III), whereas the differences between the PR values did not reach this level of statistical significance.

*DNA flow cytometric analysis*

The frequency of metastases was significantly lower ($P < 0.0001$) among the DNA-diploid cases (29%) than in the aneuploid group (57%). When DNA ploidy was related to the site of first metastasis no consistent correlation between the proportion of DNA-aneuploid tumours was found (Table IV). Furthermore, no significant difference was found between the DNA-diploid and DNA-aneuploid cases in terms of their survival curves (Figure 5).

*Histological malignancy grading*

Histological grading has been considered, despite its limitations, as a valuable biological criterion for prognosis in primary breast cancer. Therefore the evaluation of histological grading was included in this study to define its importance to recurrent breast cancer.

**Table IV** DNA ploidy in 226 cases of primary and recurrent breast cancer

| | | | *Recurrences (%)* | | |
|---|---|---|---|---|---|
| | *No. of cases* | *NED (%)* | *Viscera* | *Bone* | *Soft* |
| Diploid | 89 | 71 | 9 | 4 | 16 |
| Aneuploid | 137 | 43 | 22 | 10 | 25 |

**Table III** Oestrogen and progesterone receptor concentrations related to first site of recurrence in breast cancer

| | *ER<3 fmol* | | *ER 3–100 fmol* | | *ER>100 fmol* | | | *Mean* | *Median* |
|---|---|---|---|---|---|---|---|---|---|
| *Site* | *No.* | *%* | *No.* | *%* | *No.* | *%* | *Total* | *value* | *value* |
| Bone | 17 | 20 | 27 | 18 | 24 | 42 | 68 | 127 | 46.0 |
| Soft tissues | 35 | 41 | 76 | 50 | 23 | 42 | 134 | 67 | 11.5 |
| Viscera | 34 | 39 | 48 | 32 | 9 | 26 | 91 | 43 | 8.0 |
| $P < 0.0001$ | | | | | | | | | |
| | *PR<10 fmol* | | *PR 10–100 fmol* | | *PR>100 fmol* | | | *Mean* | *Median* |
| | *No.* | *%* | *No.* | *%* | *No.* | *%* | *Total* | *value* | *value* |
| Bone | 28 | 21 | 17 | 20 | 23 | 32 | 68 | 109 | 27.0 |
| Soft tissues | 56 | 41 | 45 | 52 | 33 | 46 | 134 | 75 | 27.0 |
| Viscera | 51 | 38 | 24 | 28 | 16 | 22 | 91 | 94 | 5.0 |
| $P < 0.04$ | | | | | | | | | |

**Figure 5** Survival after first recurrence by ploidy of the primary tumour. Diploid tumours (**d**), aneuploid tumours (**a**) ($P$ = not significant).

**Figure 6** Survival after first recurrence by histological malignancy grade. Grade I (**a**), grade II (**b**), grade III (**c**) ($P = 0.0001$).

The evaluation of histologial grading was possible in 234 out of the 313 cases included in this study. Only 18 grade I tumours were found, whereas 76 cases were recorded as grade II. The vast majority of cases with recurrent breast cancer, 140 in total, belong to grade III. The post-relapse survival curves, when related to histological grading, show that patients with grade I tumours survive longest after recurrence of their disease, whereas the shortest survival after relapse was found for patients having grade III lesions.

The survival of patients with grade II tumours lay in between. The differences between the survival curves were statistically significant ($P = 0.0001$) (Figure 6).

*Multivariate analysis*

A multivariate analysis of the prognostic variables was intended. However, the proportion of single variables used in this material does not fit well with the Cox's proportional hazard model, mainly because of the unequal number of cases for the evaluation of each variable. The number of cases for the ploidy multivariate analysis was considerably smaller than the number of other variables and therefore ploidy was not included in the multivariate analysis. To obtain a greater number of cases for this analysis, histological grades I and II were arbitrarily pooled as were bone and viscera groups for first site of recurrence. After these modifications to the material the multivariate analysis shows that histological grading, recurrence-free survival time and site of primary recurrence are the most important predictive variables in recurrent breast cancer (Table V).

### Discussion

The present analysis of 313 cases of breast cancer which subsequently developed metastases shows that patients with soft tissue recurrences had a significantly better prognosis than those with bone metastases, and that patients with visceral metastases had the worst prognosis. The better prognosis for soft tissue recurrences is in general attributed to the earlier detection and better management or less aggressiveness of such recurrences, whereas liver metastases, which are more common among histologically aggressive tumours, lead to a rapid progression of the disease (Cutler *et al.*, 1969; Hietanen, 1987). Another cause for the shorter survival of cases with visceral metastases may be the rapid impairment of the affected organs and their poor response to treatment (Hietanen, 1987).

A longer recurrence-free period and a better overall survival rate have been observed among breast cancer patients with postive ER and PR (Bishop *et al.*, 1979; Osborne *et al.*, 1980; Blamey *et al.*, 1980; Blanco *et al.*, 1984; Thorpe *et al.*, 1986). This finding has been regarded as an indication of a better response to hormonal therapy among such patients. A better survival rate was also seen here for the ER-positive patients than for the ER-negative ones, regardless of the site of recurrence. ER status also emerges as a prognostic variable upon recurrence of the disease, as described by Clark *et al.* (1987). However, the value of PR as a prognostic determinant in recurrent breast cancer is nevertheless shown here to be stronger than that of ER.

The disease-free interval following the first recurrence is of great significance for survival (Cutler *et al.*, 1969; Aberzik, 1986; Hietanen, 1987), although there are also some reports to the contrary (Rosenman & Perrone, 1984). The present patients, with a recurrence detected during the first year after diagnosis of the primary disease, fared extremely poorly compared with those whose recurrence was detected during the second year or later following diagnosis of their disease.

A number of authors have suggested that ER-positive tumours are more likely to recur in the bone and ER-negative ones in the viscera and the brain (Singhakowinta *et al.*, 1976; Stewart *et al.*, 1981; Clark *et al.*, 1987). The present results also point to a greater proportion of ER and PR-negative patients having recurrences in the lung and liver while those ER and PR-positive patients more often had recurrences in bone. Furthermore, visceral metastases appeared more frequently in the group with no ER or a low ER content, while bone recurrences were found in the group in which the highest ER values were found.

This relationship was less marked for PR. The relationship between sex steroid hormone receptors and the site of the

**Table V** Multivariate analysis in recurrent breast cancer

| | *Relative risk of death* | *95% confidence interval of relative risk* | *P* |
|---|---|---|---|
| Histological malignancy grading | 1.7 | 1.2–2.4 | 0.0039 |
| Recurrence free survival | 0.98[a] | 0.97–0.99 | 0.0029 |
| Site of first recurrence | 2.4 | 1.7–3.3 | 0.0000 |

[a]Continuous covariate.

first recurrence is highly statistically significant in terms of the median concentrations of ER and PR in relation to the site of the first recurrence. The higher the concentrations of ER and PR, the more likely it is that the first recurrence will be in bone, whereas the lower the concentrations are, the higher the possibility of visceral metastases. Accordingly, the relationship between sex steroid hormone receptors and the first site of metastases not only depends on the presence or absence of receptors but seems to be closely dependent on the amounts of these receptors present.

DNA ploidy evaluation of the primary tumour has proved to be a useful indicator for predicting the prognosis in patients with breast cancer (Hedley *et al.*, 1984; Cornelisse *et al.*, 1987; Kallioniemi *et al.*, 1987). The proportion of patients in this study with DNA diploid tumours who developed metastases in the course of their disease is significantly lower (29%) than that of patients with DNA aneuploid tumours (57%). Measurement of the DNA ploidy of the primary tumour is less useful, however, when related to the first site of recurrence, for the proportions of cases with DNA diploid tumours and DNA aneuploid tumours were similar regardless of the site of the first recurrenece. Nor do the survival curves show any differences between the groups.

Histological grading emerges in this study as a predictive variable, as was expected. However, the distribution of histological grades in the data set is not equal. The majority of the cases had histological grade III tumours, which have a high malignant potential, and account for the majority of the recurrent cases in breast cancer.

Multivariate analysis points to the same variables, histological grading, disease free-interval and site of recurrence, as the most valuable predictive factors in recurrent breast cancer. However, female sex steroid receptors also appear to have a specific role in recurrent breast cancer. Ploidy assessment, on the contrary, contributes less to the understanding of recurrent breast cancer.

### References

ABERZIK, W.J., SILVER, B., HENDERSON, C., CADY, B. & HARRIS, J.R. (1986). The use of radiotherapy for treatment of isolated locoregional recurrence of breast carcinoma after mastectomy. *Cancer*, **58**, 1214.

BISHOP, H.M., BLAMEY, R.W., ELSTON, C.W. & HAYBITTLE, J.L. (1979). Relationship of estrogen receptor status to survival in breast cancer. *Lancet*, **ii**, 283.

BLANCO, G. (1980). Prognostic factors affecting 5-year survival in breast cancer. *Acta Univ. Oulvensis Radiologica*, **2**, 3.

BLANCO, G., ALAVAIKKO, M., OJALA, A. & 5 others (1984). Estrogen and progesterone receptors in breast cancer. Relationships to tumour histopathology and survival of patients. *Anticancer Res.*, **4**, 383.

BLAMEY, R.W., BISHOP, H.M., BLAKE, J.R.S. & 5 others (1980). Relationships between primary breast tumour receptor status and patient survival. *Cancer*, **46**, 2765.

BLOOM, H.J.G. & RICHARDSON, W.W (1957). Histological grading and prognosis in breast cancer. A study of 1409 cases of which 359 have been followed for 15 years. *Br. J. Cancer*, **11**, 359.

CLARK, G.M., SLEDGE, W. Jr., OSBORNE, C.K. & MCGUIRE, W.L. (1987). Survival from first recurrence: relative importance of prognostic factors in 1,015 breast cancer patients. *Clin. Oncol.*, **5**, 55.

CORNELISSE, C.J., VAN DE VELDE, C.J.H., CASPERS, R.J.C., MOOLENAAR, A.J. & HERMANS, J. (1987). DNA ploidy and survival in breast cancer patients. *Cytometry*, **8**, 225.

CUTLER, S.J., ASIRE, A. & TAYLOR, S. (1969). Classification of patients with disseminated cancer of the breast. *Cancer*, **24**, 861.

DAVIDSON, N.E. & LIPPMAN, M.E. (1984). *Prognostic Factors in the Treatment of Metastatic Breast Cancer with Chemotherapy*. NCI: Lederle.

DEVITT, J.E. (1971). The enigmatic behavior of breast cancer. *Cancer*, **27**, 12.

DISTEFANO, A., YONG YAP, H., HORTOBAGYI, G.N. & BLUMENSCHEIN, G.R. (1979). The natural history of breast cancer patients with brain metastases. *Cancer*, **44**, 1913.

FEY, M.F., BRUNNER, K.W. & SONNTAG, R.W. (1981). Prognostic factors in metastatic breast cancer. *Cancer Clin. Trials*, **4**, 237.

FISHER, B., BAUER, M., WICKERHAM, D.L. & 2 others (1983). Relation of number of positive axillary nodes to the prognosis of patients with primary breast cancer. *Cancer*, **52**, 1551.

FISHER, E., SASS, R. & FISHER, B. (1984). Pathologic findings from the national surgical adjuvant project for breast cancers (protocol no. 4). *Cancer*, **53**, 712.

FISHER, E.R., REDMOND, C. & FISHER, B. (1980). *Pathology Annual, Part I, Vol. 15 E*. Appleton-Century-Crofts: New York.

HEDLEY, D.W., RUGG, C.A., NG, A.B.P. & TAYLOR, I.W. (1984). Influence of cellular DNA content on disease-free survival of stage II breast cancer patients. *Cancer Res.*, **44**, 5395.

HIETANEN, P. (1987). Recurrence of breast cancer. Academic dissertation, University of Helsinki.

HIETANEN, P., MIETTINEN, M. & MÄKINEN, J. (1986). Survival after first recurrence in breast cancer. *Eur. J. Cancer Clin. Oncol.*, **22**, 913.

KALLIONIEMI, O.P., BLANCO, G., ALAVAIKKO, M. & 4 others (1987). Tumour DNA ploidy as an independent prognostic factor in breast cancer. *Br. J. Cancer*, **56**, 637.

KALLIONIEMI, O.-P., BLANCO, G., ALAVAIKKO, M. & 5 others (1988). Improving the prognostic value of DNA flow cytometry in breast cancer by combining DNA index and s-phase fraction. *Cancer*, **62**, 2183.

KAMBY, C., ROSE, C., IVERSEN, H., HOLM, N.V., ANDERSEN, K.W. & THORPE, S.M. (1986). Pattern of metastases in human breast cancer in relation to estrogen receptor status. *Anticancer Res.*, **6**, 107.

KAPLAN, F.L. & MEIER, P. (1958). Nonparametric estimation from incomplete observations. *J. Am. Stat. Assoc.*, **53**, 457.

MANTEL, N. (1966). Evaluation of survival data and two new rank order statistics arising in its consideration. *Cancer Chemother. Rep.*, **50**, 163.

MÖRZ, R., FRANSCESCONI, M., SCHEMPER, M., RAINER, H., JAKESZ, J. & MOSER, K. (1982). The value of prognostic parameters for the stratification of advanced breast cancer patients. *J. Cancer Res. Clin. Oncol.*, **102**, 289.

NASH, C.H., JONES, S.T., MOON, T.E., DAVIS, S.L. & SALMON, S.E. (1980). Prediction of outcome in metastatic breast cancer treated with Adriamycin combination chemotherapy. *Cancer*, **46**, 2380.

NIKKANEN, T.A.V. (1981). Recurrence of breast cancer. *Acta Chir. Scand.*, **147**, 239.

OSBORNE, C.K., YOCHMOWITZ, M.G., KNIGHT, W.A. & MCGUIRE, W.L. (1980). The value of estrogen and progesterone receptors on the treatment of breast cancer. *Cancer*, **46**, 2884.

PATER, J., MORES, D. & LOEB, M. (1981). Survival after recurrence of breast cancer. *Can. Med. Assoc. J.*, **124**, 1591.

ROSENMAN, J. & PERRONE, T. (1984). The metastasis free interval following curative treatment for breast cancer. *Int. J. Radiat. Oncol. Biol. Phys.*, **10**, 63.

SCARFF, R.W. & TORLONI, H. (1986). *Histological Typing of Breast Tumors*. WHO: Geneva.

SINGHAKOWINTA, A., POTTER, H.G., BUROKER, T.R. & 3 others (1976). Estrogen receptor and natural course of breast cancer. *Am. Surg.*, **183**, 84.

STEWART, J.F., KING, R.J.B., SEXTON, S.A., MILLIS, R.R., RUBENS, R.D. & HAYWARD, J.L. (1981). Oestrogen receptors, sites of metastatic disease and survival in recurrent breast cancer. *Eur. J. Cancer*, **17**, 449.

THORPE, S.M., ROSE, C., RASMUSSEN, B.B. & 6 others (1986). Steroid hormone receptors as prognostic indicators in primary breast cancer. *Breast Cancer Res. Treat.*, **7**, 91.

VALAGUSSA, P., BRAMBILLA, C. & BONADONNA, G. (1979). Advanced breast cancer: are the traditional stratification parameters still of value when patients are treated with combination chemotherapy? *Eur. J. Cancer*, **15**, 565.

VIHKO, R., JÄNNE, O., KONTULA, K. & SYRJÄLÄ, P. (1980). Female sex steroid receptor status in primary and metastatic breast carcinoma and its relationship to serum steroid and peptide hormone levels. *Int. J. Cancer*, **26**, 13.

WALLGREN, A.C., SILFVERSWÄRD, C. & EKLUND, G. (1976). Prognostic factors in mammary carcinoma. *Acta. Radiol.*, **151**, 16.